# Development of a nutrient‐dense complementary food using amaranth‐sorghum grains

**DOI:** 10.1002/fsn3.367

**Published:** 2016-04-22

**Authors:** Judith Kanensi Okoth, Sophie Atieno Ochola, Nicholas K. Gikonyo, Anselimo Makokha

**Affiliations:** ^1^ Department of Food Science and TechnologyJomo Kenyatta University of Agriculture and TechnologyP.O. Box 62000‐00200NairobiKenya; ^2^ Department of Foods, Nutrition and DieteticsKenyatta UniversityP.O. Box 43844‐00100NairobiKenya; ^3^ Department of Pharmacy and Complementary/Alternative MedicineKenyatta UniversityP.O. Box 43844‐00100NairobiKenya

**Keywords:** Antinutrients, grain amaranth, nutrients, processing, stunting

## Abstract

Thin porridge from cereals and starchy tubers is a common complementary food in Sub Saharan Africa. It may be high in antinutrients, low in energy, and nutrient density hence inadequate in providing infants' high energy and nutrients requirements per unit body weight. Consequently, undernourishment levels among children under 5 years are high. Therefore, there is need to avail nutrient‐dense complementary foods especially for children in low‐resource settings. The study was aimed at developing a nutrient‐dense complementary food from amaranth and sorghum grains. Amaranth grain, a pseudocereal, though rarely used as a complementary food in Kenya has a higher nutritional quality than other staples. Plant‐based foods are known to have high levels of antinutrients. Steeping and germination were used to reduce the levels of antinutrients and enhance the bioavailability of minerals in the grains. Various steeped and germinated amaranth and sorghum grains formulations were made to find the ratio with the highest nutrient content and lowest antinutrient levels. The 90% amaranth‐sorghum grains formulation had significantly (*F* = 32.133, *P* < 0.05) higher energy (5 kcal per g on dry weight basis) than the other formulations and a protein content of 14.4%. This is higher than the estimated protein needs from complementary foods even for a 12–23 months child of low breast milk intake (9.1 g/d). Antinutrients could not be detected which could imply enhanced nutrient bioavailability. Therefore, a nutrient‐dense complementary food product was developed from steeped and germinated amaranth and sorghum grains with 90% amaranth grain. In ready to eat form, it would give an energy content of 1.7 kcal per g (dilution of 1:2 amaranth‐sorghum flour to water) and 1.2 kcal per g (dilution of 1:4 amaranth‐sorghum flour to water). It can be used as a nutrient‐dense complementary food and for other vulnerable groups.

## Introduction

According to Kenya Demographic Health Survey 2014 (KNBS and ICF Macro 2015), the proportion of stunted and wasted children in Kenya was 26% and 4%, respectively. Children aged 6–11 months had the highest wasting rates of 7%. These children fail to attain their full potential of growth and development, suffer long‐term deprivation of energy, nutrients, and consequently chronic protein energy malnutrition (PEM), often accompanied by micronutrient deficiencies. The period 6–24 months of age is one of the most critical periods in the growth of the infant. At this age, their demand for nutrients relative to their body size is high. However, there are limitations in the quality and quantity of available complementary foods (Dewey and Adu‐Afarwuah 2008; Owino et al. 2008; Imdad et al. 2011).

Some of the challenges during this period include the use of plant‐based complementary foods that are too bulky for the weanling with a tiny stomach to eat the necessary quantities that provide adequate nutrients and energy to meet their requirements. Besides plant‐based foods notably unrefined cereals, legumes, and nuts contain high levels of phytates and at times polyphenols (Gibson and Ferguson 2008). These components vary in the degree to which they inhibit iron and zinc absorption (Gibson and Ferguson 2008; WHO/FAO 2004). Therefore, the children who mainly rely on these unprocessed foods may develop PEM and micronutrient deficiencies. Hence, there is a need to develop nutrient‐dense complementary foods for such children.

**Figure 1:**
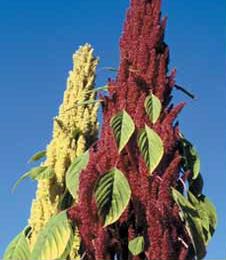
Amaranth grain.

There is currently, a lot of interest in the nutritional value of amaranth plant, whose leaves are eaten as a vegetable in many parts of Kenya (Mlakar et al. 2010). Amaranth seeds (Fig. 1) contain more protein than most grains such as wheat, maize, rice, and sorghum (Mlakar et al. 2010; Mburu et al. 2012). Further, they contain relatively high levels of micronutrients especially iron, phosphorus, magnesium, vitamin A and E (Kauffman and Weber 1990; Alemu 2008). Besides, amaranth grain is recommended for infants because of its high protein digestibility, absorption, and retention by the baby's body system (Kauffman and Weber 1990). In addition, amaranth has satisfactory lysine and tryptophan contents by FAO/WHO standards. This makes it a valuable complement to staples like maize or sorghum which are limited in amino acid lysine and tryptophan, respectively. Amaranth seed is, however, deficient in the amino acid leucine. Kauffman and Weber (1990) reported threonine as the limiting amino acid in amaranth seeds. However, amaranth grain is not commonly consumed in Kenya, though it could give a nutrient‐dense complementary food. In this study, sorghum grain was used to complement amaranth grain because it is a drought‐tolerant traditional staple cereal that is widely utilized in Kenya.

A number of traditional food processing technologies such as germination and lactic acid fermentation have been proposed as ways to improve nutrient density of complementary foods and reduce antinutrients (Gibson and Hotz 2007). This study, therefore, aimed at developing a nutrient‐dense high‐quality complementary food from amaranth and sorghum grains through processing (steeping and germination).

## Methodology

### Amaranth‐sorghum grains product development (processing)

The ingredients were processed to optimize the nutrient density while reducing the antinutrient content. Then product formulation was done followed by nutrient and antinutrient content determination.

#### Preparation of raw materials

**Figure 2:**
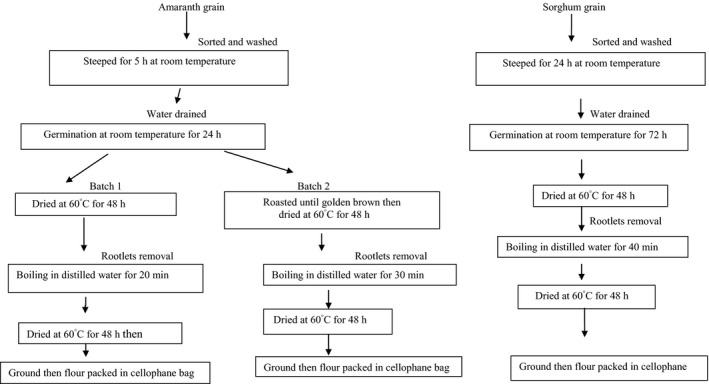
Processing of amaranth and sorghum grains for product development.

One batch, each of amaranth and sorghum grains were prepared as shown in Figure 2. The grains were sorted, washed, steeped, and germinated at room temperature. They were then dried in an oven at 60°C for 48 h and the rootlets were removed. The grains were then boiled until soft for (30 min), dried, and ground into flour. A second batch of amaranth grain was roasted immediately after germination to find out if the nutrient content would be better than when it was only boiled after germination. The flours were stored in sealed cellophane bags in a cold room at 10°C.

#### Formulation of the complementary food from amaranth‐sorghum flours

Amaranth grains that were steeped for 5 h and germinated for 24 h as well as sorghum grains steeped for 24 h and germinated for 72 h, were ground into flour, and then mixed in various ratios as shown in Table 1 (Okoth et al. 2011). The product formulation was aimed at providing adequate energy and protein content while minimizing antinutrients content. The formulations were then stored in cellophane bags at 0°C prior to laboratory analyses.

**Table 1:** Formulation of nutrient‐dense complementary food

Formulations of the grains	Amaranth:sorghum ratios
Amaranth boiled + sorghum boiled	90:10
Amaranth boiled + sorghum boiled	80:20
Amaranth boiled + sorghum boiled	70:30
Amaranth boiled + sorghum boiled	60:40
Amaranth roasted, boiled + sorghum boiled	90:10
Amaranth roasted, boiled + sorghum boiled	80:20
Amaranth roasted, boiled + sorghum boiled	70:30
Amaranth roasted, boiled + sorghum boiled	60:40

### Determination of energy and protein content of the formulations

The gross energy content was determined using a bomb calorimeter (C400) (Gallenkamp, Loughbrough, UK) while the protein content was determined using the semi‐micro kjeldahl method (AOAC 1995; Method 20.87‐32.1.22).

### Determination of the nutritional profile of the developed amaranth and sorghum grains product

Moisture and fat contents were determined by hot air drying (AOAC 1995, Method 925. 10‐32.1.03) and Soxhlet extraction (AOAC 1995; Method 920.85‐32.1.13) methods, respectively. Fatty acids were converted to methyl esters using 5% HCl in methanol (w/v), followed by extraction with hexane (Ichihara and Fukubayashi 2009). The methyl esters were then separated and quantified by gas chromatography and flame ionization detection (Ciftci et al. 2009). Crude Fiber content was quantified using the Henneberg–Stohmann method (AOAC 1995; Method 920.86‐32.1.15). Total mineral content was determined by dry ashing method (AOAC 1995; Method 923.03‐32.1.05). The total iron and zinc contents were determined by wet digestion and with an atomic absorption spectrophotometer (AAS) (AOAC 1995; Method 970.12). Product carbohydrate content was calculated by difference method as described by FAO (2010), Shahnawaz et al. (2009) and James (1995) given below:

Total carbohydrate in 100 g of food=100‐weight in g of (Protein+Water+Ash+Fat)

where g = grams.

The HCl extractable iron and zinc in the product were determined as described by Mbithi‐Mwikya et al. (2000). Iron and zinc availability in the product was determined by the method described by Svanberg et al. (1993) and modified by Matuschek et al. (2001). Sugars were separated and quantified using AOAC (1995), method 980.13. *β*‐carotene was determined using UV‐visible spectrophotometric method as described by Srivastava and Sanjeev (1998).

### Determination of the antinutrient content of the amaranth‐sorghum product

The tannin content of the amaranth‐sorghum product was determined using the modified vanillin–HCl method (Makokha et al. 2002 adapted from Price et al. 1978 and Burns 1963). The phytate content was determined by the Camire and Clydesdale (1982) method. In vitro‐protein digestibility was determined by the Mertz et al. (1984) method.

## Results and Discussion

### Formulation of the amaranth‐sorghum (complementary) food product

The various formulations that were made, their moisture, energy, and protein content are given in Table 2. The mean energy content of the formulations was 4.5 kcal with, a minimum of 3.5 kcal and a maximum of 5 kcal. The mean protein content of the formulations was 14.7% with a minimum of 12.9% and a maximum of 17.4%. The formulation with germinated and boiled amaranth grain and germinated and boiled sorghum grain in the ratio of 90:10 had the highest energy (5.0 ± 0.2 kcal per g). This is higher as compared to energy obtained from 1 g of both carbohydrate (4 kcal) and protein (4 kcal). The energy content of the formulation with 90% amaranth grain was significantly higher than that of the other formulations (*F* = 32.133, *P* < 0.05). However, there were no significant difference between the protein content of the 90% amaranth formulation and the other formulations (*F* = 2.319, *P* = 0.127). On the basis of energy content, the 90:10 amaranth to sorghum content formulation was chosen as the best for the complementary food. The moisture content was used to express the results on dry weight basis (dwb).

**Table 2:** The mean of moisture, energy, and protein content of the formulations (dwb)

Nutrients	Formulation amaranth:sorghum ratio	Moisture content (%)	Energy (kcal per g dwb)	Protein content (% dwb)
ABSI	90:10	4.8 ± 0.3	5.0 ± 0.2^1^	14.4 ± 0.6
ABS2	80:20	4.8 ± 0.3	4.7 ± 0.1^1^	14.9 ± 0.7
ABS3	70:30	5.2 ± 0.0	4.5 ± 0.2^1^	14.2 ± 0.3
ABS4	60:40	4.4 ± 0.0	3.5 ± 0.2	14.0 ± 0.1
ARBS1	90:10	2.7 ± 0.3	4.8 ± 0.1^1^	17.4 ± 0.0
ARBS2	80:20	2.9 ± 0.1	4.7 ± 0.2^1^	15.0 ± 0.2
ARBS3	70:30	3.2 ± 0.7	4.6 ± 0.1^1^	14.1 ± 0.0
ARBS4	60:40	3.8 ± 0.4	4.4 ± 0.1	12.9 ± 0.0

*N* = 3. Values are means ± standard deviation of triplicate analysis. dwb, dry weight basis. Key: ABS1, Amaranth boiled + Sorghum, steeped, germinated, boiled; ABS2, Amaranth boiled + Sorghum, steeped, germinated, boiled; ABS3, Amaranth boiled + Sorghum, steeped, germinated, boiled; ABS4, Amaranth boiled + Sorghum, steeped, germinated, boiled; ARBS1, Amaranth roasted, boiled + sorghum steeped, germinated, boiled; ARBS2, Amaranth roasted, boiled + sorghum steeped, germinated, boiled; ARBS3, Amaranth roasted, boiled + sorghum steeped, germinated, boiled; ARBS4, Amaranth roasted, boiled + sorghum steeped, germinated, boiled. Significantly different *P* < 0.05.The energy content of the formulation with 90% amaranth grain was significantly higher than that of the other formulations (F=32.133, *P* < 0.05).

“Energy value should be viewed in light of its density in the ready to eat form of a complementary food because of differences in the viscosity in foods leading to bulk densities” (Mbithi‐Mwikya et al. 2000). According to Wardlaw and Kessel (2002), a child at 6 months requires 700 kcal of energy per day. To meet this need, a 6‐month‐old child will need to take two cups (350 mL each) of the complementary food in a day with the assumption that 1 mL/g of the food provides about 1 kcal. A child between 1 and 3 years requires 1022 kcal of energy per day (Burgess, A., and P, Glasauer. 2004). Therefore, they would need to take two cups of 300 mL of the amaranth‐sorghum food in a day with a dilution of 1:2 (Amaranth‐sorghum product to water).

The formulation with a ratio of 90 (steeped, germinated, roasted, and boiled amaranth grain):10 (germinated and boiled sorghum grain) had the highest protein content. However, the differences in protein content among the different formulations were not significant. The formulation chosen for the product had a protein content of 14.4%. This is higher than the estimated protein needs from complementary foods even for a 12–23‐month‐old child of low breast milk intake (9.1 g/d). The formulation chosen contains 28.8 g protein per 1000 kcal. This is considerably higher than that in the World Health Organization/Food and Agriculture Organization (WHO/FAO) report on protein requirements (WHO 2007), which recommended a minimum of 6.9% energy contributed by protein.

### Nutritional properties of developed amaranth‐sorghum product and the effect of processing on the nutrients

The proximate composition of the product is given in Table 3. The carbohydrate content was 71.4%. It provides an energy content of 1.2 kcal per 1 g wet weight basis. The product provides 28.8 g protein per 1000 kcal of energy. It had a glucose content of 14.8 g per 100 g, fructose 9.3 g per 100 g, and sucrose content of 12.3 g per 100 g. Michaelsen et al. (2008) reported that the most important dietary mono‐ and disaccharides are glucose, fructose, lactose, and sucrose (sugar). These sugars are good sources of energy and will typically increase the energy density of a diet (Michaelsen et al. 2008).

**Table 3:** Nutritional profile of the developed complementary food on dwb

Nutrient	Quantity
Moisture %	5.2 ± 0.0
Energy kcal per g	5.0 ± 0.2
Protein %	14.4 ± 0.3
Fat %	6.8 ± 0.4
Ash %	2.2 ± 0.1
Crude fiber %	3.0 ± 0.1

Values are means ± standard deviation of triplicate analysis. dwb, dry weight basis.

The proximate composition of fortified blended foods (FBF) commonly used by World Food Programme (WFP) to rehabilitate moderately malnourished children is given in Table 4. The FBF nutritional value was compared with that of the developed food product (Table 3). The developed complementary food has a higher energy content compared to the FBF. The developed amaranth‐sorghum product protein (14.4%) provides 28.8 g protein per 1000 kcal of energy. This is higher than the protein per kcal in FBF (Table 4). Further, there was no indigestible protein detected in the product. The inability to detect indigestible protein in the product can be attributed to the decrease in the level of antinutrients (Singh et al. 1982; Kataria et al. 1989; Mbithi‐Mwikya et al. 2000).

**Table 4:** Nutritional value of commonly used food aid commodities

	Energy (kcal per 100 g)	Fat (MJ/100 g)	Fat (E %)	Protein (g per 100 g)	Protein (E %)
Corn Soy blend	3.8	1.6	14.2	18.0	18.9
Wheat Soy blend	3.7	1.55	14.6	20.0	21.1
Corn Soy milk	3.8	1.6	14.2	20.0	21.1

In the study, no fat was added to the product, as is usually done in commercially blended foods. Usually in FBFs, the oil is added at the time of distribution to minimize use of oil for other purposes. Fat tends to reduce the shelf life of food. The energy content supplied by fat in the amaranth‐sorghum product was 12.2% of the total energy in the product. Linoleic acid (LA) was the highest in amount compared with the other fatty acids in the product as given in Table 5 while lauric acid (0.34 mg/100 g) was the least. The essential fatty acids in the product provided 7.4% energy (E %) of the total energy on dwb. The n‐3 polyunsaturated fatty acids (PUFA) provided by the developed product was 1.8 E %.

**Table 5:** Essential fatty acids and fatty acid profile of the complementary food

Fatty acid composition	Abbreviation	Quantity (mg per 100 g dwb)
Caprylic acid	C8:0	0.68
Lauric acid	C12:0	0.034
Myristic acid	C14:0	47.6
Palmitic acid	C16:0	115.6
Oleic	C18:1	176.8
Linoleic acid	C18:2	231
Linolenic acid	C18:3	10.2
Stearic acid	C18:0	11.6

Values are means ± standard deviation of triplicate analysis. dwb, dry weight basis.

There are two types of essential fatty acids, the n‐6 and the n‐3 PUFA, which in most diets are provided by vegetable oils in the form of LA (25 C18:2n‐6) and *α*‐linolenic acid (ALA, C18:3n‐3), respectively. According to Wulf et al. (2008) and Michaelsen et al. (2009), the fat intake of young children (1–2 years) should have a quality that provides 5–10 E % as essential fatty acids including at least 1 E % of n‐3 PUFA and have a ratio of n‐6 to n‐3 PUFA between 3 and 9. For complementary feeding of children who are well nourished, a level of 30–45 fat E % was recommended, including the fat from breast milk (Dewey and Brown 2003). Several authors have recommended that fat should supply 30–45% of energy intake for children less than 2 years of age (Michaelsen and Jorgensen 1995; Mbithi‐Mwikya et al. 2000; Wardlaw and Kessel 2002).

**Figure 3:**
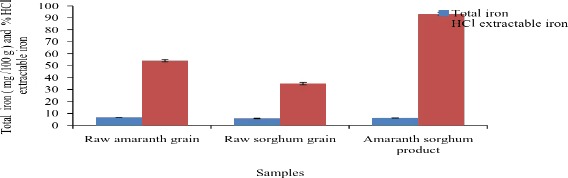
Total iron and HCl extractable iron content of the product and its ingredients (dry weight basis).

The crude fiber was less than 5%. It is recommended that insoluble fibers should be present in low amounts in the diet because they increase bulk and reduce gastrointestinal transit time (Michaelsen et al. 2009). Dietary fibers may reduce energy intake through a suppressing effect on appetite and they may increase fecal losses of energy due to reduced absorption of fat and carbohydrate (Aggett et al. 2003; Michaelsen 2009). The amaranth‐sorghum product had a total iron content of 6 mg/100 g, a value slightly lower than that of raw amaranth grains (6.6 mg per 100 g) (Fig. 3). It had a higher HCl extractability of iron than its ingredients (93.3%) and its bioavailable iron was 5.5 mg per 100 g (dwb) (11.2 mg per 1000 kcal).

**Figure 4:**
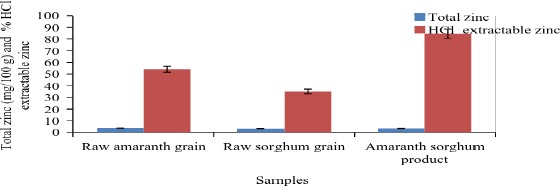
The total and HCl extractable zinc content in the product and its ingredients.

The amaranth‐sorghum product had a total zinc content of 3.2 mg per 100 g as given in Figure 4. The HCl extractable zinc in the food product was 84% which was higher than both its ingredients. Also, it had 2.8 mg per 100 g (dwb) (5.6 mg per 1000 kcal) bioavailable zinc.

The developed amaranth‐sorghum food product had a higher availability of iron and zinc compared to raw amaranth and sorghum grains. For a food‐based approach and for most formulated diets, it is important that the basic ingredients be such that the iron is more available (Golden 2009). According to Wardlaw and Kessel (2002), the recommended daily intake of iron and zinc for children 7–12 months is 11 mg per day (mg/d) while those 1–3 years it is 7 mg/d. HCl extractability of minerals and trace elements under simulated gastric conditions is an indicator of bioavailability in foods (Antony and Chandra 1998). The improvement in in vitro iron and zinc bioavailability observed in this study could be attributed to a decrease in phytic acid among other antinutrients.

The complementary food contained 4.2 *μ*g per 100 g of *β*‐carotene (7.1 I.U of vitamin A; 0.35 retinol activity equivalents per 100 g) on dwb. It also provided 0.7 retinol activity equivalents/1000 kcal. The recommended daily allowance of vitamin A is 400, 500, and 300 retinol equivalents for children 0–6 months, 7–12 months, and 1–3 years, respectively. The complementary food, therefore, will not supply the estimated needs of vitamin A for children below 23 months.

### Antinutritional components of the amaranth‐sorghum product (dwb)

Raw amaranth grain had a tannin content of 0.8% CE while sorghum had a tannin content of 24.9% CE. No tannins could be detected in the product. Phytates levels (inositol hexaphosphate) were 7.9 and 252 mg per 100 g in raw amaranth and sorghum grains, respectively. No phytates were detected in the developed complementary food. The most important antinutritional food constituent in diets in low‐income countries in terms of negative nutritional impact is phytate, primarily contributed by cereal staples and secondarily by legumes and other plant foods (Michaelsen et al. 2009). Phytate forms insoluble complexes with a range of nutrients and thereby inhibits the absorption of protein and minerals in particular iron, zinc, and calcium. One of the most widespread groups of polyphenols with antinutritional properties is soluble tannins (Michaelsen et al. 2009). The antinutritional effect of polyphenols is complex formation with iron and other minerals, and precipitation of protein, which reduces absorbability. The fact that the antinutrients in the product could not be detected in the steeped and germinated grain implies that minerals and nutrients usually bound by them could be more bioavailable.

## Conclusion

A complementary food from amaranth and sorghum grains with an energy density of 5 kcal per g sufficient to meet the recommendations for children 6–23 months of age at 2–3 servings per day was developed. The food product was also low in antinutrients and therefore of correspondingly high nutrient availability and digestibility.

## Conflict of Interest

None declared.
